# Cation Substitution in High‐Entropy Layered Double Hydroxide Driving D‐Band Center Tuning for Oxygen Evolution Reaction

**DOI:** 10.1002/advs.202517723

**Published:** 2025-11-20

**Authors:** Pinnan Li, Jingwei Li, Christophe Colbeau‐Justin, David Berardan, Mohamed Nawfal Ghazzal

**Affiliations:** ^1^ Université Paris‐Saclay, CNRS UMR 8000 Institut de Chimie Physique Orsay 91400 France; ^2^ National Energy Key Laboratory for New Hydrogen‐Ammonia Energy Technologies Foshan Xianhu Laboratory Foshan China; ^3^ Université Paris‐Saclay, UMR 8182 CNRS Institut de Chimie Moléculaire et des Matériaux d'Orsay (ICMMO) Orsay 91405 France

**Keywords:** d‐band center, high‐entropy layered double hydroxide, OER, precious‐metals‐free electrocatalyst

## Abstract

The development of electrocatalysts with optimized intermediate adsorption and low energy barriers is crucial for the oxygen evolution reaction (OER). In this work, the d‐band center position of high‐entropy layered double hydroxides (HE‐LDHs) is modulated by substituting Mg^2^⁺ sites with Fe^2+^, Cu^2+^, Co^2+^, and Ni^2+^. It is demonstrated that the d‐band center position relative to the Fermi level is modified, reaching an optimal energy in (FeCuCoNi)_6_Al_2_‐LDH. The nature of the incorporated transition metals significantly influenced OH^−^ adsorption kinetics and reduced the overpotential for OER by 55%, compared to native LDH. The stepwise substitution of Mg^2^⁺ by Fe^2^⁺ particularly induces charge carrier transfer, switching into Faradaic processes favorable to the enhancement of OER kinetics. This work provides an effective approach that allows decreasing the OER overpotential through adjusting the position of the d‐band center, and suggests that d‐band tuning via multication insertion can directly shift the material toward an optimal binding strength region, which underlies the observed performance.

## Graphical abstract

The d‐band center position of layered double hydroxide is adjusted via multication insertion in the octahedral site. The optimal position of the d‐band, near the Fermi level, enhances OER performance through optimal OH^−^ adsorption kinetics, resulting in a 55% reduction in overpotential compared to the native catalyst.

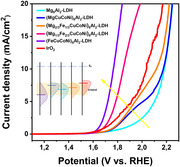

## 1 Introduction

The discovery of entropy stabilized crystalline structure has catalyzed a paradigm shift in materials science. First reported for high entropy alloys,^[^ ^1^ ^]^ it was later extended to oxides in 2015 by Rost and coworkers.^[^ ^2^ ^]^ High‐entropy oxides (HEOs), which combine multiple cations into a single homogeneous phase, offer researchers unprecedented flexibility in tuning structural and electronic properties.^[^ ^3^ ^]^ This versatility arises from their ability to sustain various cations in multiple configurations, thereby creating a highly customizable material framework. HEOs allow researchers to explore a vast array of tunable properties. Consequently, various structural crystalline structures such as rocksalt‐type structures,^[^ ^4^ ^]^ single‐phase CaF_2_ structures,^[^ ^5^ ^]^ perovskites,^[^ ^6^ ^]^ and spinels,^[^ ^7^ ^]^ have been synthesized, each exhibiting remarkable properties suitable for diverse applications.

The structural and compositional flexibility of HEOs, particularly in the rational manipulation of their electronic structure,^[^ ^8^, ^9^ ^]^ offers extensive possibilities for designing and optimizing efficient electrocatalysts for oxygen evolution reactions. Indeed, electrocatalytic water splitting is the most advanced technology, enabling the substitution of fossil energy resources and reducing carbon emission issues.^[^ ^10^ ^]^ While the HER is relatively fast and requires a lower overpotential in most cases, the oxygen evolution reaction (OER) is kinetically sluggish.^[^ ^11^, ^12^ ^]^ OER involves the transfer of four electrons during the formation of O─O bond, making it the rate‐determining step in the overall water splitting process. The sluggish kinetics are mainly controlled by surface processes on the catalyst. The basics of understanding water oxidation kinetics involve the adsorption/desorption of water at the surface of the catalyst. To gain deeper insights into this surface‐controlled kinetics, it is essential to examine the electronic structure of the catalyst. The Fermi level (E_F_) is the highest occupied energy level at absolute zero. At room temperature, electrons follow the Fermi–Dirac distribution, and E_F_ corresponds to the energy where the occupation probability is 50%. This makes E_F_ a fundamental reference point in photoelectron spectroscopy and electronic structure analysis, particularly when evaluating the position of the d‐band center. The d‐band center, first proposed by Nørskov and coworkers, represents the average energy level of electrons in the d‐orbital of transition metals and serves as a theoretical descriptor for catalytic activity.^[^ ^13^ ^]^ From this perspective, it has been shown that the adsorption and desorption behavior of reaction intermediates on catalyst surfaces is closely related to the position of the d‐band center.^[^ ^14^ ^]^ A higher d‐band center lies closer to E_F_, increasing orbital overlap with adsorbates and potentially improving catalytic performance — provided that the intermediate binding strength remains within an optimal range. Thus, adapting the distance between the d‐band center position (E_d_) and the Fermi level (E_F_) to reach optimal OER kinetic reaction is an important parameter to investigate. For example, the original E_d_ of Ni_2_P was increased by the Fe substitution strategy, promoting the adsorption of oxygen‐formed intermediates.^[^ ^15^ ^]^ Similarly, Fe doping Ni_3_Ge_2_O_5_(OH)_4_ serpentine modulated the d‐band state, affecting the formation of intermediates during the OER.^[^ ^16^ ^]^ Besides, the d‐band center theory has been applied to noble metals.^[^ ^17^, ^18^, ^19^ ^]^ However, its use for HE‐layered double hydroxide (HE‐LDH) is scarcely reported. It is speculated that doping the inorganic oxide structure induces the movement of the d‐band center, thereby reducing the band gap required for charge transfer.^[^ ^20^, ^21^ ^]^ Therefore, it is important to tailor the E_d_ of HE‐LDH and understand its effect on OER. The versatility of HE‐LDH not only offers the opportunity to select transient metals with variable valence electrons, making them a platform where d‐band center theory could be applied, but it can also be obtained easily at low temperatures under relatively mild conditions.^[^ ^22^ ^]^ Additionally, HE‐LDH nanoparticles have shown promise as electrocatalysts once coupled to noble metals or grown on a Ni foam substrate.^[^ ^23^, ^24^ ^]^ The Ni‐supported HE‐LDH showed lower OER overpotential and Tafel slope.^[^ ^24^ ^]^ However, the intrinsic electrocatalytic properties of HE‐LDH remain related to the catalyst performance of the Ni foam substrate.

In this work, we report the improvement of the intrinsic catalytic activity of noble metal‐free HE‐LDH for OER by adjusting the d‐band center position through the substitution of Mg cation sites compared to the native MgAl‐LDH. As shown in Table S1 (Supporting Information), a series of HE‐LDHs were prepared by systematically substituting Mg^2^⁺ with transition metals Fe, Cu, Co, and Ni, while maintaining Al^3^⁺ as the trivalent cation. In the final composition, complete replacement of Mg^2^⁺ resulted in the formation of (FeCuCoNi)_6_Al_2_‐LDH, a five‐metal system with extensive cationic mixing. The nature of the incorporated transition metals significantly influenced the d‐band center position relative to the Fermi level, thereby modulating OH^−^ adsorption kinetics and reducing the overpotential for OER. The configurational entropy (ΔS_mix_) was calculated by considering all metal cations (M^2^⁺ and M^3^⁺) occupy the same crystallographic site in the LDH lattice. Indeed, it has been demonstrated that there is no long‐range M^2+^‐M^3+^ ordering in Mg_6_Al_2_‐LDH, which would render such ordering highly unlikely in multi‐cationic compositions. It leads to ΔS_mix_ values—ranging from 1.602 R to 1.722 R (Table S1, Supporting Information) — demonstrating that these compositions exceed the widely accepted threshold (ΔS_mix_ ≥ 1.5 R) for high‐entropy classification.

## 2 Results and Discussion

### 2.1 Structure and Morphology Characterizations

**Figure 1:**
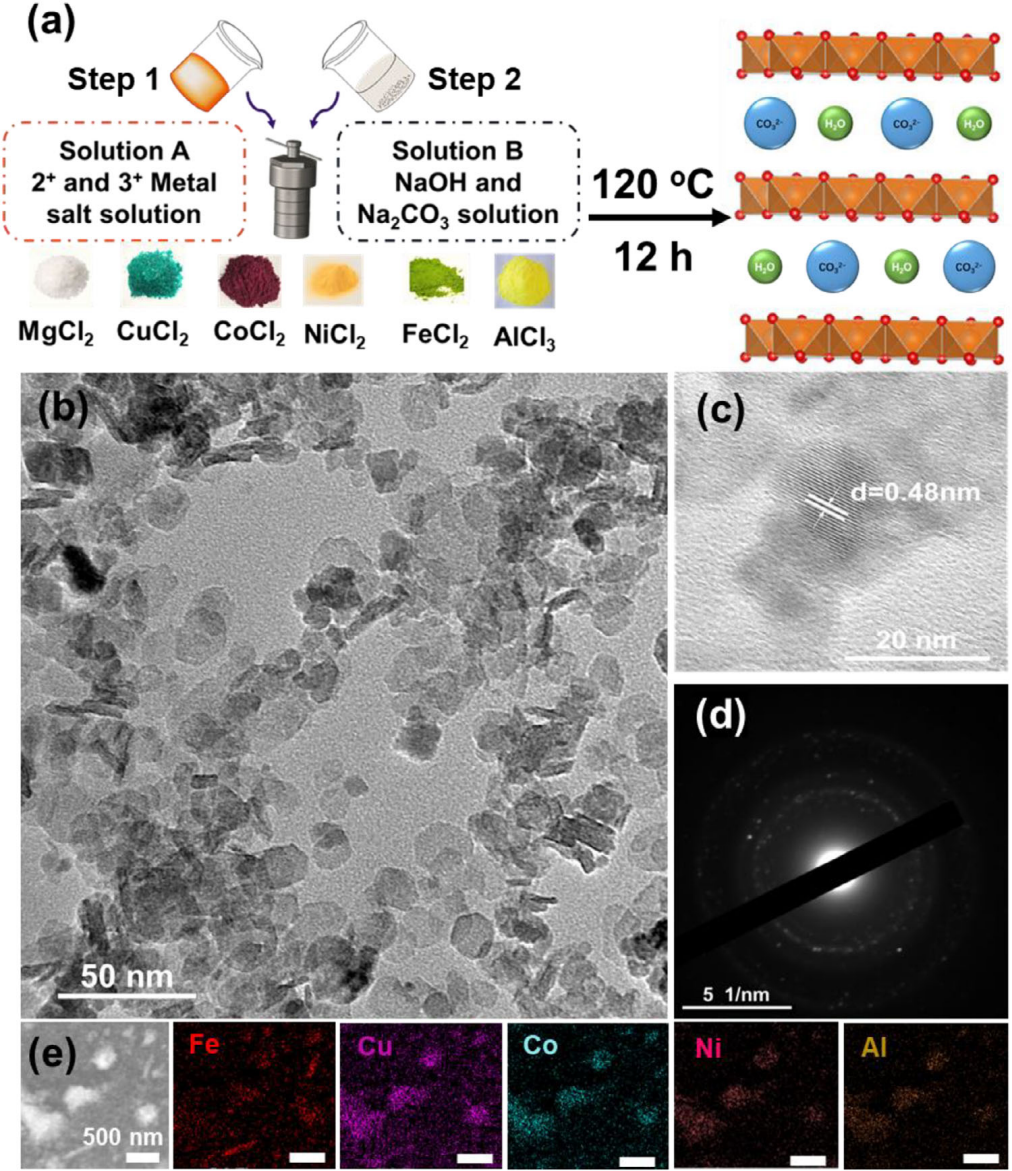
a) Scheme diagram of the synthesis process. b) TEM figure of (FeCuCoNi)_6_Al_2_‐LDH (50 nm). c) The interplanar spacing of brucite. d) SAED of (FeCuCoNi)_6_Al_2_‐LDH. e) The EDS mapping of (FeCuCoNi)_6_Al_2_‐LDH.

The high‐entropy and reference samples were synthesized using a hydrothermal method, as described in **Figure** **1**a. Briefly, the precursors of variable metal cations were introduced into the Teflon vial of the autoclave in stoichiometric proportions according to the nominal compositions (valences of +2 and +3) and heated at 120 °C for 12 h under alkaline conditions. The morphology of the obtained materials is observed using Transmission Electron Microscopy (TEM). The morphology of the Mg_6_Al_2_‐LDH reference sample is predominantly spherical, exhibiting a wide size distribution ranging from 20 to 200 nm. The average size is estimated to be ≈70 nm, as shown in Figure S1 (Supporting Information). Figure S1 (Supporting Information) shows the energy‐dispersive spectroscopy (EDS) analysis of the sample, indicating the presence of mainly Mg, Al, and O (the Cu signal corresponds to the copper grid used for TEM analysis). The substitution of Mg atoms by several cations to obtain the HE‐LDHs leads to a size reduction and shapes the nanoparticle's morphology into a hexagonal form. The (FeCuCoNi)_6_Al_2_‐LDH sample exhibited a 2D hexagonal‐like nanostructure, with a size range of 10–20 nm, as shown in Figures 1b and S2a,b (Supporting Information). HRTEM revealed a fringe characteristic of crystalline structure with an interplanar spacing of 0.48 nm (Figure 1c). The interplane distance is consistent with the presence of a brucite layer, as reported in previous studies.^[^ ^25^, ^26^ ^]^ This observation confirms that the high‐entropy (FeCuCoNi)_6_Al_2_‐LDH sample retains its layered double hydroxide architecture, indicating that the incorporation of multiple transition metal cations does not disrupt the long‐range ordering of the lamellar structure. The EDS chemical mapping of the chosen region displayed the presence of these metals (FeCuCoNi)_6_Al_2_‐LDH (Figure 1d). The morphology of (MgCuCoNi)_6_Al_2_‐LDH is also observed by TEM, and the results displayed in Figure S3 (Supporting Information) indicate that there is no change in the morphology or the size of HE‐LDH nanoparticles after the cations’ substitution.

**Figure 2:**
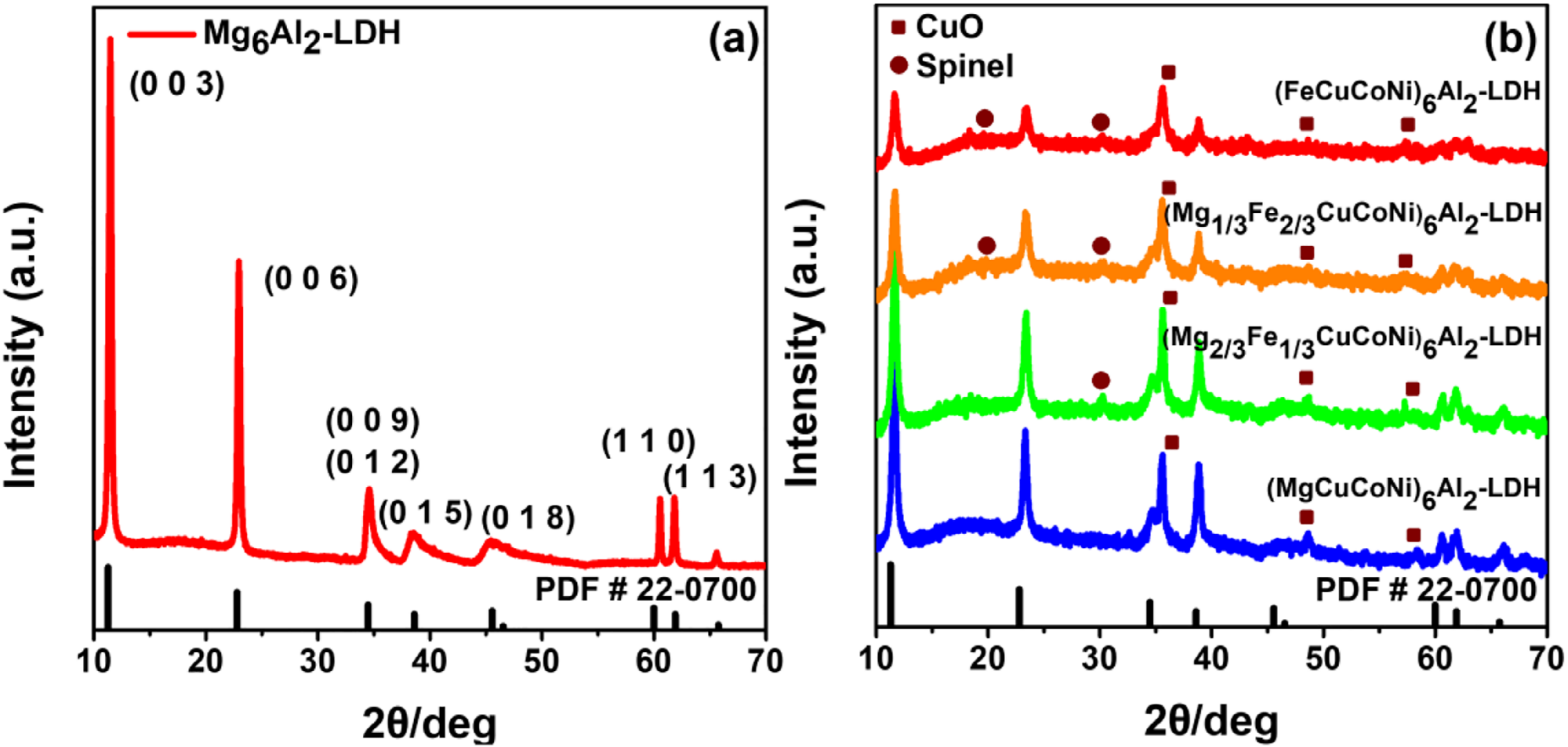
XRD spectra of a) the reference sample Mg_6_Al_2_‐LDH, b) HE‐LDHs samples.

The crystalline structure is determined by means of X‐ray diffraction analysis, and the results are presented in **Figure** **2**. XRD patterns indicated that the main phase is Mg_6_Al_2_‐layered double hydroxide. The sharp and symmetric diffraction peaks at 11.47°, 22.95°, 60.54°, and 61.82° are assigned to the (003), (006), (110), and (113) diffraction planes, respectively. The pattern showed broad and asymmetric peaks assigned to (009), (015), and (018). This may indicate different stoichiometries or the presence of significant strain due to a broad particle size distribution ranging from 20 to 200 nm, as shown in Figure S1b (Supporting Information).^[^ ^27^, ^28^, ^29^, ^30^ ^]^ The absence of clear diffraction peaks that could be assigned to Mg(OH)_2_ or Al(OH)_3_ indicates that the main crystalline phase is MgAl‐layered double hydroxide.^[^ ^31^ ^]^ The lattice parameters estimated from the refinement are a = b = 3.061 Å and c = 23.34 Å (Figure S4, Supporting Information). The high‐entropy samples exhibit XRD patterns that conform to the hydrotalcite‐like structure; however, they have lower crystallinity, as indicated by the broader peaks (Figure 2b). In addition, compared with the Mg_6_Al_2_‐LDH reference, high entropy samples exhibit slightly shifted diffraction peaks toward higher angles. Besides, additional diffraction peaks indicating the formation of CuO and spinel secondary phases are observed. The small amount of spinel phase might be Fe_3_O_4_ because it has a low formation free energy, and part of Fe^2^⁺ is oxidized to Fe^3^⁺.^[^ ^32^, ^33^, ^34^ ^]^ Although a CuO impurity phase is detected in all HE‐LDH samples, its signal intensity remains the same and consistent among the compositions, indicating a comparable composition in all LDH structures.

**Figure 3:**
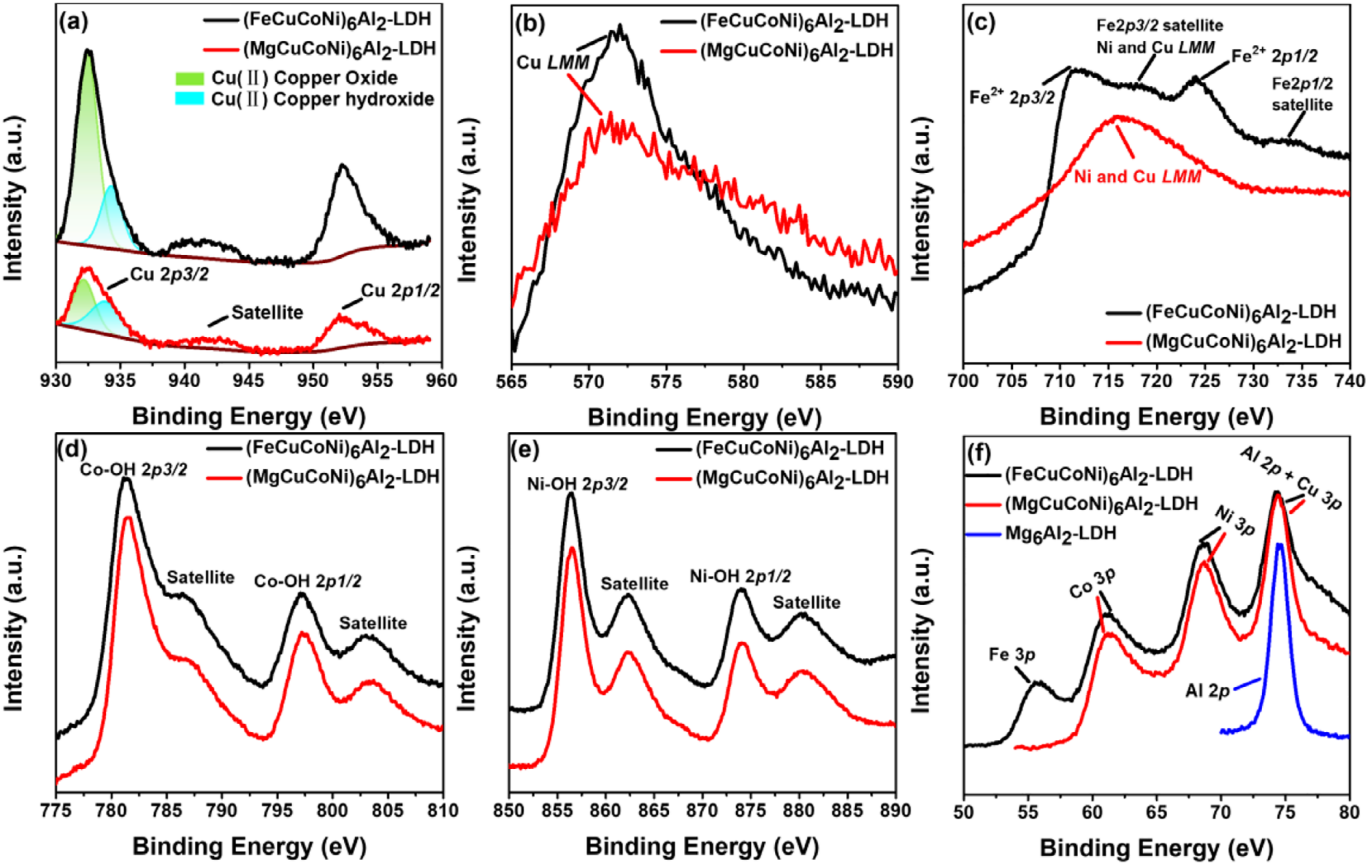
XPS spectra of a) Cu (II), b) Cu *LMM*, c) Fe2*p*, d) Co 2*p*, e) Ni 2*p*, and f) 3*p* orbital peaks of different metals compared between (MgCuCoNi)_6_Al_2_‐LDH, (FeCuCoNi)_6_Al_2_‐LDH, and Mg_6_Al_2_‐LDH.

X‐ray photoelectron spectroscopy (XPS) was employed to determine the chemical oxidation state of different elements in HE‐LDHs, and the results are presented in **Figure** **3**. The survey spectra shown in Figure S5a (Supporting Information) revealed the presence of Mg, Fe, Cu, Co, Ni, Al, O and C in (MgCuCoNi)_6_Al_2_‐LDH and (FeCuCoNi)_6_Al_2_‐LDH samples, while the Mg_6_Al_2_‐LDH reference sample contains mainly Mg, Al, O and C. High‐resolution C 1s peak of Mg_6_Al_2_‐LDH reference sample (Figure S5b, Supporting Information) can be deconvoluted into four peaks. The main peak at 284.7 eV corresponds to C─C bands from adventitious carbon. A shoulder at 286.2 eV is assigned to C─O bonds in CO_3_^2^^−^, which appears at 288.9 eV. A weak signal at 283.3 eV, likely arising from metal and carbon interactions, is only found in the Mg_6_Al_2_‐LDH reference.^[^ ^35^ ^]^ The O1s peak could be deconvoluted into three components. The high‐resolution peak O 1s is located at 531.3 eV (Figure S5c, Supporting Information) for all samples. The lattice oxygen (M─O) is centered at 530.0 eV, and carbonates are 532.7 eV. The high‐resolution XPS spectra of Cu 2*p* in (FeCuCoNi)_6_Al_2_‐LDH and (MgCuCoNi)_6_Al_2_‐LDH showed peaks with binding energy located at 932 eV (Cu 2*p3/2)* and 952 eV (Cu 2*p1/2*) (Figure 3a), which is characteristic of Cu^2^⁺.^[^ ^36^ ^]^ The Cu 2*p3/2* peak can be deconvoluted into two peaks centered at 932.5 and 934.4 eV assigned to Cu^2+^ in CuO and copper hydroxide, respectively.^[^ ^37^ ^]^ The broad satellite peak at 941.5 eV further confirmed the presence of Cu^2+^.^[^ ^37^ ^]^ The peak at 571 eV in the Cu LMM spectra (Figure 3b) also demonstrates the presence of copper hydroxide. The formation of CuO in HE‐LDH is likely due to the strong Jahn–Teller distortion of Cu^2^⁺ (3d⁹) in octahedral coordination, which introduces local lattice strain and promotes Cu segregation.^[^ ^38^, ^39^ ^]^ The existence of Fe was evidenced by the presence of the Fe 2*p* peak in (FeCuCoNi)_6_Al_2_‐LDH. The peaks of Fe 2*p3/2* and 2*p1/2* have binding energies centered at ≈711.3 and 724.7 eV, in agreement with Fe^2+^ in LDH.^[^ ^40^, ^41^ ^]^ Nevertheless, the deconvolution of the Fe 2*p* peak cannot be performed as it overlaps with the Ni and Cu LMM peaks, which interfere with precise spectral deconvolution in this range.^[^ ^42^, ^43^ ^]^ However, the presence of Fe^3+^ cannot be neglected, likely due to the presence of a secondary spinel structure. Additionally, the Fe 3*p* orbital signal is clearly observed in Figure 3f, further confirming the presence of iron. The Co and Ni 2*p* spectra (Figure 3d,e) exhibit no noticeable changes in the binding energy of main peaks and satellite peaks after Fe incorporation. As predicted by the Irving–Williams series, Co^2^⁺ and Ni^2^⁺ offer greater crystal field stabilization energies (CFSE) than Fe^2^⁺ and do not experience Jahn–Teller distortion.^[^ ^44^, ^45^, ^46^ ^]^ This makes them more likely to coordinate with hydroxyl ligands in a regular [M(OH)_6_] octahedral environment.^[^ ^47^ ^]^ These results indicate that Fe substitution does not significantly affect the local chemical states of Co and Ni in the LDH structure.

### 2.2 Electrochemical Performance Evaluation of OER Catalysts

**Figure 4:**
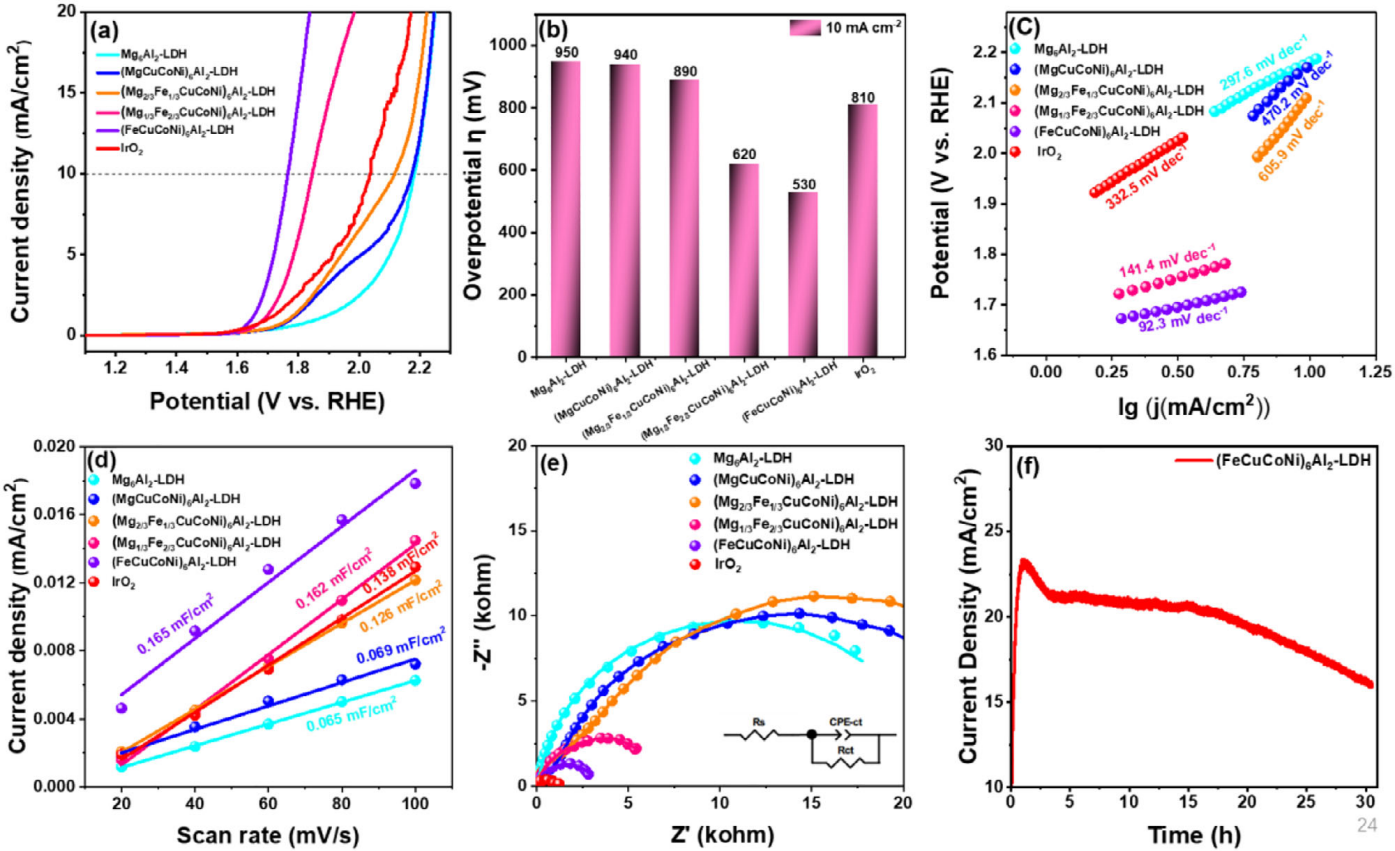
a) LSV curves with 95% iR correction and 2 mV s^−1^ scan rate. The amount of catalyst is 0.29 mg on 1 × 1 cm^2^ carbon cloth, and the solution resistance (R_s_) is 2.50 Ω as determined by EIS. The potential after iR correction is manually adjusted according to the formula V_corrected_ = V_applied_ − iR_s_×0.95. b) Overpotential comparison at 10 mA cm^−2^ current density. c) The Tafel slope plots. d) Cdl curves. e) The EIS curves. (The applied potential is 0.5 V versus Ag/AgCl, and the frequency is 10^5^ – 10^−1^ Hz.) f) Stability test of (FeCuCoNi)_6_Al_2_‐LDH.

The effects of cation substitution and the formation of HE‐LDHs on OER performance were evaluated by linear sweep voltammetry (LSV) measurements in 1.0 M KOH electrolyte using a Nova 2.1.5 electrochemical workstation under a scan rate of 2 mV s^−1^. To minimize the impact of solution resistance, a 95% iR correction was applied, and the difference between 95% and 100% correction is shown in Figure S6 (Supporting Information). As presented in **Figure** **4**a, the HE‐LDHs samples exhibit significantly enhanced OER activity compared to the reference. The overpotential achieved at 10 mA cm^−2^ decreases as the gradual substitution of Mg by Fe is performed (Figure 4b). In particular, the catalyst with full Mg substitution exhibits the best performance, achieving even lower overpotential than commercial IrO_2_. Additionally, we have compared the electrochemical performance and structural properties of monosubstituted Mg‐LDHs with Co, Ni, Cu, and Fe with HE‐LDH catalyst. The LSV and XRD patterns of monosubstituted Mg‐LDHs with each cation are shown in Figures S7 and S8 (Supporting Information). We can observe that the crystalline structure of the layered double hydroxides remains preserved in the cases of Co_6_Al_2_‐LDH and Ni_6_Al_2_‐LDH. However, the samples with Fe and Cu showed additional diffraction patterns. Indeed, Fe_3_O_4_ spinel is formed as the main phase with LDH structure, while with Cu, the crystalline phase was dominated by CuO. Conversely, the Mg^2+^ substitution by Co^2+^ and Ni^2+^ enables crystal phase stabilization of the LDH structure. The Cu and Fe in an octahedral environment undergo a strong Jahn–Teller distortion,^[^ ^38^, ^39^ ^]^ as predicted by the Irving–Williams series, and form a secondary phase due to their low formation free energy. However, Co and Ni can provide a stable LDH network, which could allow Fe ions to better integrate the HE‐LDH structure. Additionally, the amount of these secondary phases decreases significantly in the high‐entropy material, indicating that Co and Ni may play a role as stabilizers in the LDH structure. The linear sweep voltammetry presented in Figure S8 (Supporting Information) shows the highest performance observed for (FeCuCoNi)_6_Al_2_‐LDH. The Co sample exhibits a better OER performance compared to other reference samples. The results demonstrate that the secondary phase observed in the HE‐LDH cannot be the main cause of the enhanced OER performance, further supporting our findings.

To further analyze the reaction kinetics, Tafel slopes were derived from the LSV data analysis, and the results are shown in Figure 4c. The Fe‐substituted Mg in HE‐LDHs catalyst exhibits a notably low Tafel slope of 92.3 mV dec^−1^, indicating efficient charge transfer. Compared with the reference and samples with partial substitution of Mg in HE‐LDHs, the Fe/HE‐LDHs catalyst demonstrates significantly enhanced electron transport properties. The reduced Tafel slope suggests that the rate‐determining step (RDS) in the multi‐electron OER pathway likely occurs in the later stages, involving the deprotonation of surface‐bound oxygen intermediates.^[^ ^48^, ^49^ ^]^ The electrochemical surface area (ECSA) was estimated from the double‐layer capacitance (Cdl) obtained via cyclic voltammetry (CV) at different scan rates (Figure S9, Supporting Information). As illustrated in Figure 4d, the enhanced catalyst exhibits the highest ECSA value, even surpassing that of IrO_2_, indicating a greater density of active sites. Electrochemical impedance spectroscopy (EIS) measurements provide further insights into charge transfer dynamics. As shown in the Nyquist plots (Figure 4e), the charge transfer resistance (R_ct_) of the fully Fe‐substituted high‐entropy catalyst is significantly lower than that of the other samples, as indicated by the R_ct_ values in Table S2 (Supporting Information). The lower R_ct_ indicates that HE‐LDHs materials exhibit improved electronic conductivity and efficient interfacial charge transfer. Notably, as the Fe content increases in HE‐LDHs, the R_ct_ decreases, reinforcing the correlation between composition and charge transport properties. Although IrO_2_ exhibits a lower charge‐transfer resistance, the (FeCuCoNi)_6_Al_2_‐LDH catalyst demonstrates superior OER activity, which is attributed to its larger electrochemically active surface area (ECSA) and consequently higher density of active sites. The long‐term stability of the optimized catalyst was evaluated using chronopotentiometry at 1.823 V versus RHE. As depicted in Figure 4f, the current density remained stable with negligible decay over a continuous 20 h testing period, indicating its relative stability. A gradual decrease in current density was observed beyond 20 h, which is likely attributable to leaching of the catalyst from the support during the prolonged gas evolution process, as observed during the test. The activity loss was estimated to be ≈18% after 30 h of continuous testing. These results demonstrate that the incorporation of multications and the construction of HE‐LDHs electrodes affect the conductivity and charge transfer, increasing the density of active sites and leading to an improvement in OER performance.

**Figure 5:**
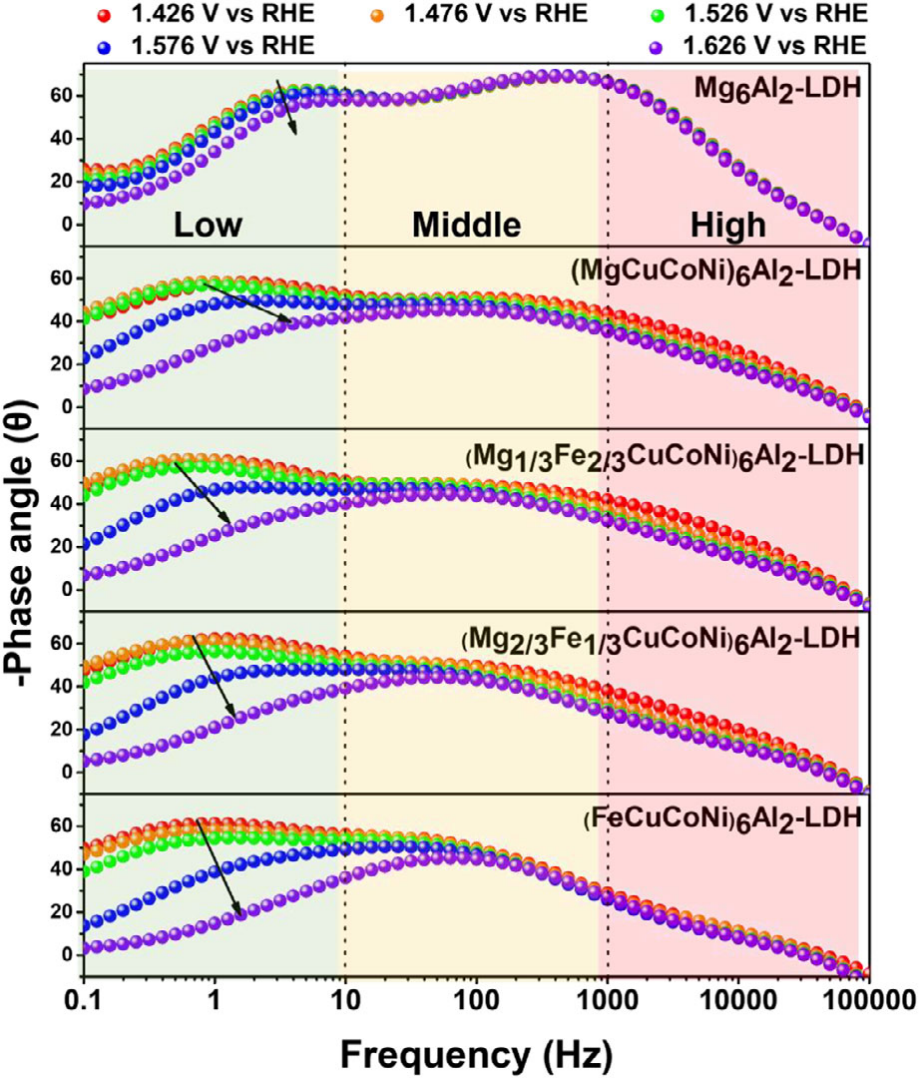
In situ Bode phase angle plots at different applied potentials (1.426–1.626 V vs RHE).

The in situ EIS Bode plots were used to study the evolution of OER kinetics for high‐entropy samples. The Bode phase plots depict the trend of phase angle variation with frequency. The results depicted in **Figure** **5** were obtained in the potential window of 1.42−1.62 V, in which the onset of the OER process occurs. Three regions can be observed, with one associated with low frequency and the other with high frequency, corresponding to the interface reaction charge transfer and the electron transfer process, respectively.^[^ ^50^, ^51^ ^]^ The phase angle in the middle‐frequency range for reference Mg_6_Al_2_‐LDH exhibits an intense peak, indicating a double‐layer capacitor behavior (≈10 – 1000 Hz, where non‐Faradaic behavior is dominant). In the applied window potential, a gradual decrease of the phase angle peak is observed for HE‐LDHs samples. The stepwise substitution of Mg^2^⁺ by Fe^2^⁺ particularly decreases the middle‐frequency peak, pointing to easier charge carrier transfer and switching into Faradaic processes (0.1‐10 Hz) with a decreasing accumulation of charges in double‐layer capacitance. It means that more charges will work in Faradaic processes. In the low‐frequency region, which means that the charge transfer process dominates this region, 0.1−10 Hz, the phase angle of (FeCuCoNi)_6_Al_2_‐LDH approaches 0° at high potentials (1.626 V). In other words, the interfacial process is almost completely dominated by charge transfer during the OER, and the resistance effect is negligible. In contrast, HE‐LDHs samples with lower or without Fe content still display a strong capacitive current due to the higher phase angle in that region, compared to Mg_6_Al_2_‐LDH. Upon increasing the applied potential from 1.426 to 1.626 V, all samples exhibit a gradual transition from capacitance‐dominated to charge‐transfer‐dominated behavior. However, HE‐LDHs (FeCuCoNi)_6_Al_2_‐LDH undergo this transition at significantly lower potentials, indicating earlier activation of the OER process due to the fastest decreasing speed of phase angle. The enhancement of OER kinetics is attributed to the fast charge transfer facilitated by the inclusion of multication in the HE‐LDHs structure.

### 2.3 OER Mechanism

**Figure 6:**
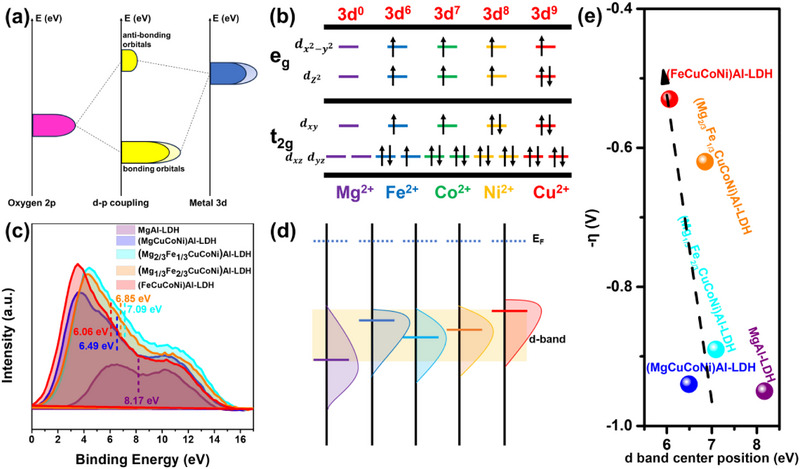
a) d‐p coupling, b) the d‐band electrons of the metals, c) the comparison of d‐band center position (the valence band center position for MgAl‐LDH), d) the flowchart of d‐band center position, e) the volcano plot of the samples.

The cation substitution in HE‐LDHs samples is expected to result in an overall shift of the d‐band center. The interaction between metal d‐orbitals and oxygen‐containing species is central to OER activity. Generally, a higher d‐electron density enhances orbital overlap, thereby promoting the adsorption and conversion of reaction intermediates (**Figure** **6**a). The position of the d‐band center relative to the Fermi level further determines the strength of this interaction. For instance, a lower energy of the d‐band center leads to weakly bound key intermediates in the OER process. On the contrary, if the energy is too high, the adsorption becomes strong, hindering their desorption and slowing down the turnover. The concept is generally known as Sabatier's principle.^[^ ^52^ ^]^ Optimal performance is thus expected when the d‐band center falls within a range that balances the adsorption/desorption processes.

The d‐band center for HE‐LDHs samples was obtained from the XPS valence band spectra using the following integral expression:^[^ ^17^, ^53^ ^]^

(1) $$\begin{array}{ccc} d-band\;center\;position & = & \int_{17eV}^{0\;eV}\left(binding\;energy(E\right)*Intensity\left(E\right)dE\\ & & /\int_{17eV}^{0\;eV}\left(binding\;energy\left(E\right)\right)dE \end{array}$$

Based on octahedral field theory,^[^ ^54^ ^]^ Figure 6b,c illustrate how changes in metal composition affect both the energy and electrons of the d‐band. For Mg_6_Al_2_‐LDH, which contains no d‐electrons, the valence band center position is located far below the Fermi level (set at 0 eV in XPS), at ≈8.17 eV. This suggests very limited interaction between the electronic states and adsorbates. Partial substitution of Mg^2^⁺ with Fe, Co, Ni, and Cu leads to a progressive upward shift of the d‐band center to 7.1 eV ((Mg_2/3_Fe_1/3_CuCoNi)_6_Al_2_‐LDH) and 6.85 eV ((Mg_1/3_Fe_2/3_CuCoNi)_6_Al_2_‐LDH), suggesting a gradual shift toward a more reactive electronic structure. The Fe‐free (MgCuCoNi)_6_Al_2_‐LDH sample shows an additional upward shift to 6.5 eV, highlighting the contributions of Co^2^⁺, Ni^2^⁺, and Cu^2^⁺ to the d‐band. Nevertheless, its electrochemical performance remains lower than that of some Fe‐containing counterparts. This could be attributed to the d electrons. The combination of Fe^2^⁺ (3d⁶), Co^2^⁺ (3d⁷), Ni^2^⁺ (3d⁸), and Cu^2^⁺ (3d⁹) significantly increases the *d*‐electron population and promotes effective *d–p* orbital overlap. Figure 6d schematically illustrates how the d‐band center and electron cloud characteristics evolve across the sample series, providing an intuitive understanding of how different cation configurations influence the electronic landscape. In the enhanced high‐entropy structure, (FeCuCoNi)_6_Al_2_‐LDH, its d‐band center shifts to 6.06 eV—close to the Fermi level—and the overall d‐electron density is significantly enhanced. These features align with the best OER performance, including the lowest overpotential and smallest Tafel slope (Figure 6e), indicating the positive impact of high‐entropy design on the electronic structure and catalytic activity. Figure 6e also explores the possible existence of a Sabatier‐type relationship between d‐band center position and activity. Although the data show a general increase in performance with a more upshifted d‐band, the expected volcano‐type trend is not observed. This suggests that most samples still fall on the weak‐binding side of the curve. To fully reveal the activity–structure relationship, it would be useful to introduce compositions with stronger binding strength and see whether they define the expected performance peak. The evolution between ΔE_d_ and overpotential suggests that d‐band tuning via multication insertion can directly shift the material toward an optimal binding strength region, which underlies the observed performance.

## 3 Conclusion

In summary, a noble‐metal‐free (FeCuCoNi)_6_Al_2_‐LDH catalyst was developed by substituting Mg^2^⁺ sites with multiple transition metals (Fe, Cu, Co, Ni), delivering markedly enhanced OER activity. Native Mg_6_Al_2_‐LDH, which does not possess d‐electrons, showed the lowest catalytic activity toward the OER reaction, higher OER overpotential, and a Tafel plot. The substitution of Mg cation by other transition metals introduces d‐electrons into the band structure at different levels regarding the Mg/Fe ratio. The shifts observed in the d‐band center allow adjusting the distance to the Fermi level, which optimizes the adsorption energetics of oxygen intermediates, thereby lowering the kinetic barrier and changing the rate‐determining step. In addition, the mechanism of OER was attributed to fast charge transfer as multications were inserted into the HE‐LDHs structure. This work highlights high‐entropy engineering as a general strategy for tailoring structure–reactivity relationships in LDH‐based electrocatalysts toward efficient water oxidation.

## 4 Experimental Section

### Materials

MgCl_2_ (Sigma–Aldrich, 95%), CuCl_2_ (Sigma–Aldrich, 99%), CoCl_2_·6H_2_O (Sigma–Aldrich, 98%), NiCl_2_ (Alfa Aesar, 98%), AlCl_3_ (Thermo Scientific, 98.5%), FeCl_2_·4H_2_O (Sigma–Aldrich, 99%), IrO_2_ (Sigma‐Aldrich, 99.9%), Na_2_CO_3_ (Sigma–Aldrich, 99%), NaOH (Sigma–Aldrich, 97%), KOH (Sigma–Aldrich, 90%), Nafion (Alfa Aesar 5%)

### (MgCuCoNi)_6_Al_2_‐LDH Preparation Method

This catalyst was synthesized using a hydrothermal method. MgCl_2_ (0.0015 mol), CuCl_2_ (0.0015 mol), CoCl_2_ (0.0015 mol), NiCl_2_ (0.0015 mol), and AlCl_3_ (0.002 mol) were dissolved in 30 mL of deionized H_2_O under N_2_ protection, marked solution A, and stirred for 30 min. Meanwhile, 0.0047 mol of Na_2_CO_3_ and 0.022 mol of NaOH were dissolved in 30 mL of deionized water to obtain solution B, which was then stirred for 30 min under N_2_ protection. Then, solution B was added to solution A and strongly stirred for 30 min under N_2_, protected at room temperature. The as‐prepared mixture was transferred into a 100 mL Teflon‐lined autoclave and reacted at 120 °C for 12 h. The obtained catalyst was centrifugally separated, washed with deionized water and ethanol, and dried at 60 °C for 24 h.

### (Mg_x_Fe_1‐x_CuCoNi)_6_Al_2_‐LDH Preparation Method

The synthesis method followed the (MgCuCoNi)_6_Al_2_‐LDH one, except that Mg^2+^ was substituted by Fe^2+^. The molar proportion of Mg^2+^ and Fe^2+^ was 2:1 and 1:2, respectively, and one without Mg^2+^, ((FeCuCoNi)_6_Al_2_‐LDH). For reference, 0.006 mol of MgCl_2_ and 0.002 mol of AlCl_3_ were used to synthesize the basic MgAl‐LDH.

### X‐ray Diffraction Measurements

The XRD results were tested by a Panalytical X'Pert diffractometer with a Ge (111) incident monochromator (Cu‐Kα1 radiation) and X'celerator detector. The Rietveld refinement was performed using Fullprof software.

### X‐ray Photoelectron Spectroscopy

The X‐ray Photoelectron Spectroscopy (XPS, ThermoFisher) was studied by a Kα spectrometer, and the X‐ray source is Al Kα, 1486.6 eV. VB‐XPS (with a measurement energy step size of 0.2 eV) was used to test the valence band and calculate the d‐band center position. A value of 284.8 eV for the C 1*s* peak was used for calibration of all XPS spectra. All XPS data were treated with the Shirley background.

### Transmission Electron Microscopy

Transmission electron microscopy (TEM) images were acquired using a JEOL JEM 2100Plus microscope operating at an acceleration voltage of 200 kV. Energy dispersive X‐ray spectroscopy (EDS) was scanned in scanning TEM (STEM) mode at 200 kV. The size of the particles was estimated from the obtained images, and an average size distribution was determined from multiple images, considering ≈100–200 particles (depending on the available images).

### Electrochemical Measurements

A Nova 2.1.5 electrochemical workstation and a three‐electrode cell were used. The counter electrode was Pt, and the reference electrode was Ag/AgCl. The electrolyte solution was a 1 M KOH solution, which was bubbled with N_2_ for 30 min before testing. 5 mg catalysts, 500 µL ethanol, and 20 µL Nafion were stirred together for 24 h to make the ink used on the working electrode. 30 µL of the ink was deposited onto a 1 cm^2^ carbon cloth to form the working electrode. Double‐layer capacitance (Cdl) was obtained from the CV test at different scan rates, 20, 40, 60, 80, 100 mV/s, from 0.03 to 0.14 V (vs Ag/AgCl). Calculating by the following equation, the ECSA can be obtained by:

(2) ECSA=Cdl/Cs

where the value of Cs is 40 µF cm^−2^. The EIS was executed at 0.5 V versus Ag/AgCl. The amplitude and the range of frequency were set to 5 mV and 10^5^‐10^−1^ Hz. Linear sweep voltammetry (LSV) was measured at 2 mV s^−1^ from 0 to 1.5 V versus Ag/AgCl (95% iRs corrected manually, as shown in Figure 4a). The Tafel slope can be calculated from LSV data. The Chronoamperometry test results were obtained under a stable voltage of 1.823 V versus RHE to explore the stability of the catalyst for 110000s.

## Conflict of Interest

The authors declare no conflict of interest.

## Supporting information



Supporting Information

## Data Availability

The data that support the findings of this study are available from the corresponding author upon reasonable request.;

## References

[1] B. Cantor , I. T. Chang , P. Knight , A. Vincent , Mater. Sci. Eng., A 2004, 375, 213.

[2] C. M. Rost , E. Sachet , T. Borman , A. Moballegh , E. C. Dickey , D. Hou , J. L. Jones , S. Curtarolo , J.‐P. Maria , Nat. Commun. 2015, 6, 8485. 26415623 10.1038/ncomms9485 PMC4598836

[3] Y. Pan , J.‐X. Liu , T.‐Z. Tu , W. Wang , G.‐J. Zhang , Chem. Eng. J. 2023, 451, 138659.

[4] Z. Lun , B. Ouyang , D.‐H. Kwon , Y. Ha , E. E. Foley , T.‐Y. Huang , Z. Cai , H. Kim , M. Balasubramanian , Y. Sun , Nat. Mater. 2021, 20, 214. 33046857 10.1038/s41563-020-00816-0

[5] H.‐Z. Xiang , H.‐X. Xie , A. Mao , Y.‐G. Jia , T.‐Z. Si , Int. J. Mater. Res. 2020, 111, 246.

[6] Y. Wang , J. Liu , Y. Song , J. Yu , Y. Tian , M. J. Robson , J. Wang , Z. Zhang , X. Lin , G. Zhou , Small Methods 2023, 7, 2201138. 10.1002/smtd.202201138 36843320

[7] Z. Sun , Y. Zhao , C. Sun , Q. Ni , C. Wang , H. Jin , Chem. Eng. J. 2022, 431, 133448.

[8] T. Meng , Z. Geng , F. Ma , X. Wang , H. Zhang , C. Guan , Interdisciplinary Materials 2023, 2, 589.

[9] G. Peng , L. Hu , W. Qu , C. Zhang , S. Li , Z. Liu , J. Liu , S. Guo , Y. Xiao , Z. Gao , Interdisciplinary Materials 2023, 2, 30.

[10] Y. Gong , J. Yao , P. Wang , Z. Li , H. Zhou , C. Xu , Chin. J. Chem. Eng. 2022, 43, 282.

[11] Y. Yao , J. Lyu , X. Li , C. Chen , F. Verpoort , J. Wang , Z. Pan , Z. Kou , DeCarbon 2024, 5, 100062.

[12] J. Chen , G. Fu , Y. Tian , X. Li , M. Luo , X. Wei , T. Zhang , T. Gao , C. Chen , S. Chaemchuen , Interdisciplinary Materials 2024, 3, 595.

[13] B. Hammer , J. K. Norskov , Nature 1995, 376, 238.

[14] B. Hammer , Y. Morikawa , J. K. Nørskov , Phys. Rev. Lett. 1996, 76, 2141. 10060616 10.1103/PhysRevLett.76.2141

[15] S. Sun , X. Zhou , B. Cong , W. Hong , G. Chen , ACS Catal. 2020, 10, 9086.

[16] H. Lee , O. Gwon , K. Choi , L. Zhang , J. Zhou , J. Park , J.‐W. Yoo , J.‐Q. Wang , J. H. Lee , G. Kim , ACS Catal. 2020, 10, 4664.

[17] F. Ando , T. Gunji , T. Tanabe , I. Fukano , H. D. Abruna , J. Wu , T. Ohsaka , F. Matsumoto , ACS Catal. 2021, 11, 9317.

[18] S. J. Hwang , S.‐K. Kim , J.‐G. Lee , S.‐C. Lee , J. H. Jang , P. Kim , T.‐H. Lim , Y.‐E. Sung , S. J. Yoo , J. Am. Chem. Soc. 2012, 134, 19508. 23131009 10.1021/ja307951y

[19] V. Stamenkovic , B. S. Mun , K. J. Mayrhofer , P. N. Ross , N. M. Markovic , J. Rossmeisl , J. Greeley , J. K. Nørskov , Angew. Chem., Int. Ed. 2006, 45, 2897. 10.1002/anie.200504386 16596688

[20] Z. Wang , S. Shen , J. Wang , W. Zhong , Chemistry–A European Journal 2024, 30, 202402725. 10.1002/chem.202402725 39269324

[21] C. Chen , Z. Xu , G. Hai , W. H. Huang , C. W. Pao , H. Li , K. Jiang , N. Zhang , T. Liu , Small 2025, 21, 2407964. 10.1002/smll.202407964 39502021

[22] M. Kim , I. Oh , H. Choi , W. Jang , J. Song , C. S. Kim , J.‐W. Yoo , S. Cho , Cell Reports Physical Science 2022, 3, 100702.

[23] F. Wang , P. Zou , Y. Zhang , W. Pan , Y. Li , L. Liang , C. Chen , H. Liu , S. Zheng , Nat. Commun. 2023, 14, 6019. 37758731 10.1038/s41467-023-41706-8 PMC10533845

[24] W. Zhang , X. Zhang , Y. Song , F. Gao , Y. Zhang , Chem. Commun. 2025, 61, 7532. 10.1039/d5cc01284h 40302626

[25] S. Miyata , Clays Clay Miner. 1975, 23, 369.

[26] Clays Clay Miner. 1983, 31, 305.

[27] V. R. Constantino , T. J. Pinnavaia , Inorg. Chem. 1995, 34, 883.

[28] J. I. Langford , A. Wilson , Applied Crystallography 1978, 11, 102.

[29] R. Delhez , T. H. Keijser , E. Mittemeijer , J. Anal. Chem. 1982, 312, 1.

[30] J.‐Y. Lee , G.‐H. Gwak , H.‐M. Kim , T.‐i. Kim , G. J. Lee , J.‐M. Oh , Appl. Clay Sci. 2016, 134, 44.

[31] S. Muráth , Z. Somosi , Á. Kukovecz , Z. Kónya , P. Sipos , I. Pálinkó , J. Sol‐Gel Sci. Technol. 2019, 89, 844.

[32] E. W. Lemmon , M. O. McLinden , D. G. Friend , P. Linstrom , W. Mallard , Nist chemistry webbook. NIST standard reference database 2011, 69, 20899.

[33] J. Poonoosamy , F. Brandt , M. Stekiel , P. Kegler , M. Klinkenberg , B. Winkler , V. Vinograd , D. Bosbach , G. Deissmann , Appl. Clay Sci. 2018, 151, 54.

[34] E. Bernard , W. J. Zucha , B. Lothenbach , U. Mäder , Cem. Concr. Res. 2022, 152, 106674.

[35] E. Lewin , P. Å. Persson , M. Lattemann , M. Stüber , M. Gorgoi , A. Sandell , C. Ziebert , F. Schäfers , W. Braun , J. Halbritter , Surf. Coat. Technol. 2008, 202, 3563.

[36] H. Perrot , O. Sel , C. Debiemme‐Chouvy , K. Lafdi , M. El Rhazi , Int. J. Hydrogen Energy 2021, 46, 19926.

[37] C. Wang , S. R. Mouchet , O. Deparis , J. Li , E. Paineau , D. Dragoe , H. Remita , M. N. Ghazzal , Small 2024, 20, 2402211. 10.1002/smll.202402211 38898765

[38] Z. Rák , J.‐P. Maria , D. Brenner , Mater. Lett. 2018, 217, 300.

[39] D. Berardan , A. Meena , S. Franger , C. Herrero , N. Dragoe , J. Alloys Compd. 2017, 704, 693.

[40] X. Meng , J. Han , L. Lu , G. Qiu , Z. L. Wang , C. Sun , Small 2019, 15, 1902551. 10.1002/smll.201902551 31423746

[41] N. J. Patil , N. Makkapati , V. Deeksha , R. Vishnuraj , G. Balaji , M. Rangarajan , P. Srinivasan , Appl. Surf. Sci. 2025, 712, 164147.

[42] M. N. Ghazzal , J. Goffin , E. M. Gaigneaux , Y. Nizet , J. Taiwan Inst. Chem. Eng. 2017,71, 62.

[43] A. R. Bredar , M. D. Blanchet , A. R. Burton , B. E. Matthews , S. R. Spurgeon , R. B. Comes , B. H. Farnum , Oxygen reduction electrocatalysis with epitaxially grown spinel MnFe 2022, 12, 3577.

[44] N. Greenwood , A. Earnshaw , Chemistry of the elements, 2nd edition, Butterworth‐Heinemann, 1997.

[45] F. A. Cotton , G. Wilkinson , C. A. Murillo , M. Bochmann , Advanced inorganic chemistry, 6th edition, John Wiley & Sons, 1999.

[46] C. E. housecroft , Vib. Spectrosc. 2012, 71, 7.

[47] G. Fan , F. Li , D. G. Evans , X. Duan , Chem. Soc. Rev. 2014, 43, 7040. 25001024 10.1039/c4cs00160e

[48] N. B. Watkins , Z. J. Schiffer , Y. Lai , C. B. Musgrave , H. A. Atwater , W. A. Goddard , T. Agapie , J. C. Peters , J. M. Gregoire , ACS Energy Lett. 2023, 8, 2185. 10.1021/acsenergylett.4c00204 PMC11019637 38633999

[49] B. Zhong , D. He , R. Chen , T. Gao , Y. Wang , H. Chen , Y. Zhang , D. Wang , Phys. Chem. Chem. Phys. 2019, 21, 17517. 31380550 10.1039/c9cp03541a

[50] R. Doyle , M. Lyons , J. Electrochem. Soc. 2013, 160, H142.

[51] C. Xie , W. Chen , S. Du , D. Yan , Y. Zhang , J. Chen , B. Liu , S. Wang , Nano Energy 2020, 71, 104653.

[52] J. Hwang , R. R. Rao , L. Giordano , Y. Katayama , Y. Yu , Y. Shao‐Horn , Science 2017, 358, 751. 29123062 10.1126/science.aam7092

[53] X. Wu , F. Chen , N. Zhang , Y. Lei , Y. Jin , A. Qaseem , R. L. Johnston , Small 2017, 13, 1603387. 10.1002/smll.201603387 28151572

[54] R. G. Burns , Mineralogical applications of crystal field theory, Cambridge university press, 1993.

