# Supplementary material for: Cation Substitution in High‐Entropy Layered Double Hydroxide Driving D‐Band Center Tuning for Oxygen Evolution Reaction
Source: Adv Sci (Weinh). 2025 Nov 20;13(7):e17723. doi: 10.1002/advs.202517723 (PMC12866842; doi:10.1002/advs.202517723)
Supplement: Supplementary file 1 — Supporting Information [file ADVS-13-e17723-s001.docx]

**(Supporting Information)**

**Cation substitution in High-Entropy Layered Double Hydroxide driving d-Band Center Tuning for improved Oxygen Evolution Reaction**

Pinnan Li,^a^ Jingwei Li,^b,*^ Christophe Colbeau-Justin^a^, David Berardan,^c^ Mohamed Nawfal Ghazzal^a^*

^a^ Université Paris-Saclay, CNRS UMR 8000, Institut de Chimie Physique, Orsay, 91400, France

^b^ National Energy Key Laboratory for New Hydrogen-Ammonia Energy Technologies, Foshan Xianhu Laboratory, Foshan

^c^ Université Paris-Saclay, UMR 8182 CNRS, Institut de Chimie Moléculaire et des Matériaux d'Orsay (ICMMO), Orsay, 91405 France

e-mail: mohamed-nawfal.ghazzal@universite-paris-saclay.fr

and lijingwei@xhlab.cn

**Table S1.** The ΔSₘᵢₓ values of the high entropy samples.

| Samples | ΔS_mix_=-R∑x_i_lnx_i_ |
| --- | --- |
| (MgCuCoNi)₆Al₂-LDH | 1.602R |
| (Mg_2/3_Fe_1/3_CuCoNi)₆Al₂-LDH | 1.722R |
| (Mg_1/3_Fe_2/3_CuCoNi)₆Al₂-LDH | 1.722R |
| (FeCuCoNi)₆Al₂-LDH | 1.602R |


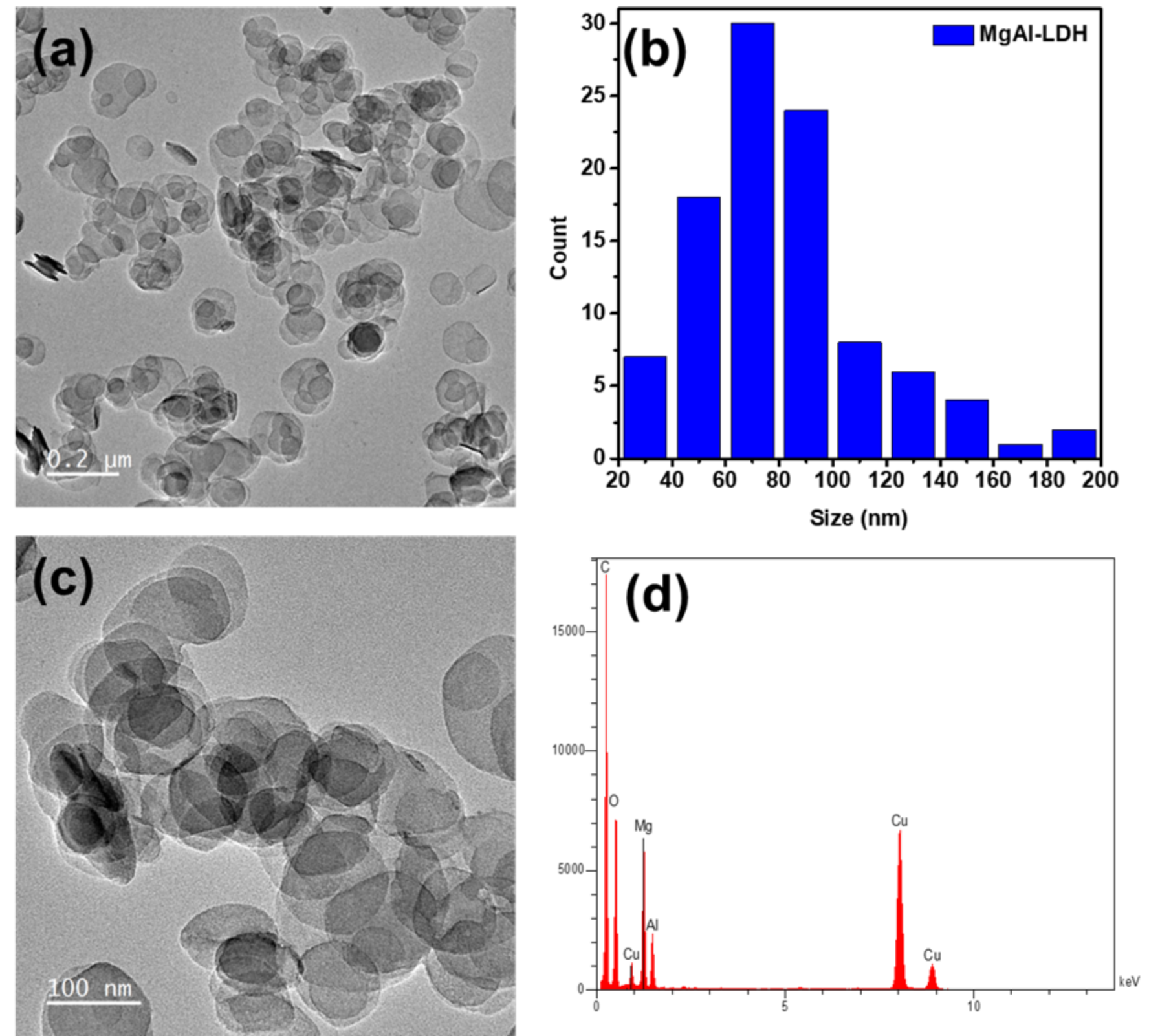


**Figure S1.** (a) TEM figure of Mg_6_Al_2_-LDH (200 nm), (b) the size count figure, (c) The TEM figure of Mg_6_Al_2_-LDH (100nm), (d) The EDS mapping of Mg_6_Al_2_-LDH.


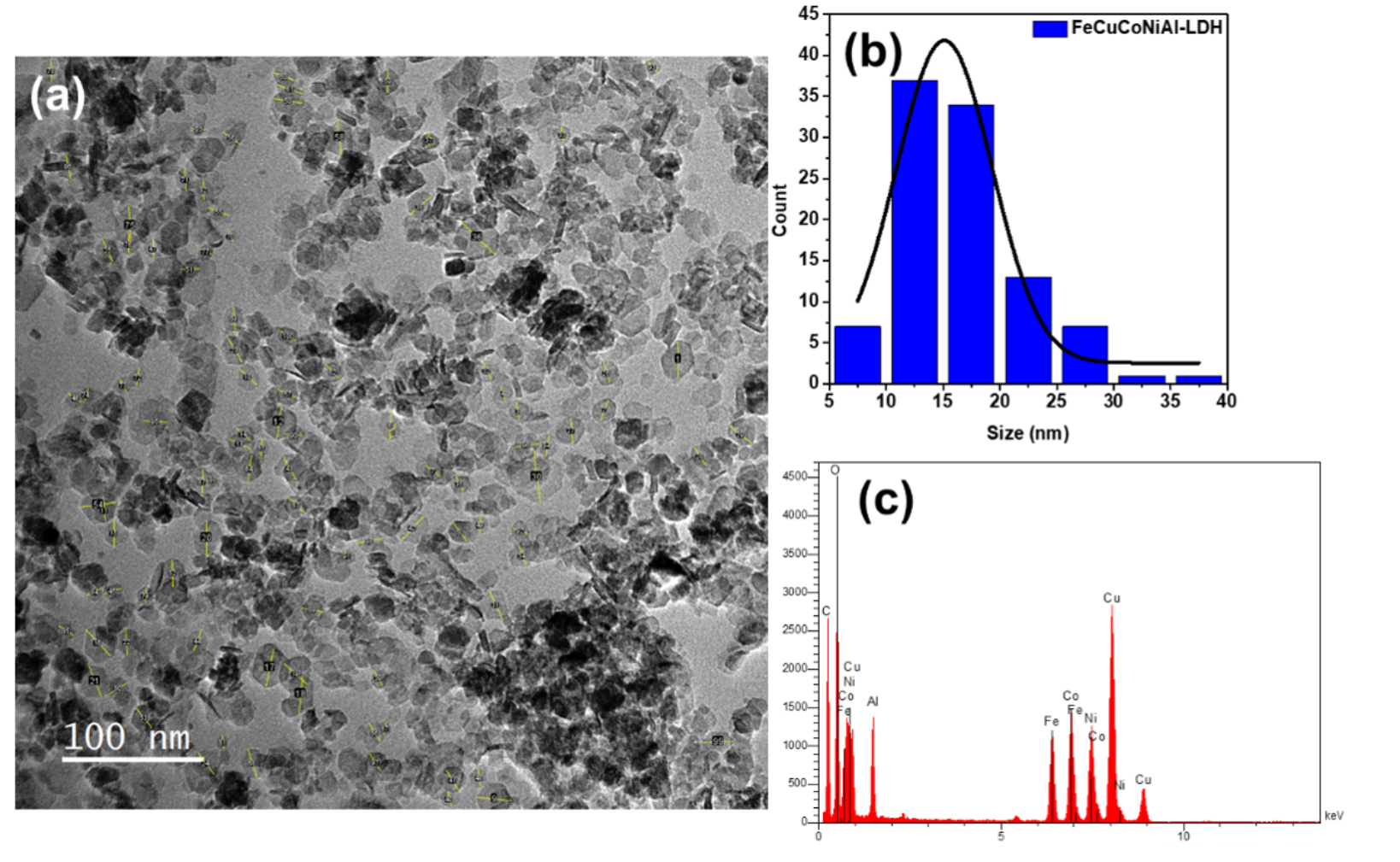


**Figure S2.** (a) TEM figure of (FeCuCoNi)_6_Al_2_-LDH (100 nm), (b) the size count figure, (c) The EDS mapping of (FeCuCoNi)_6_Al_2_-LDH.


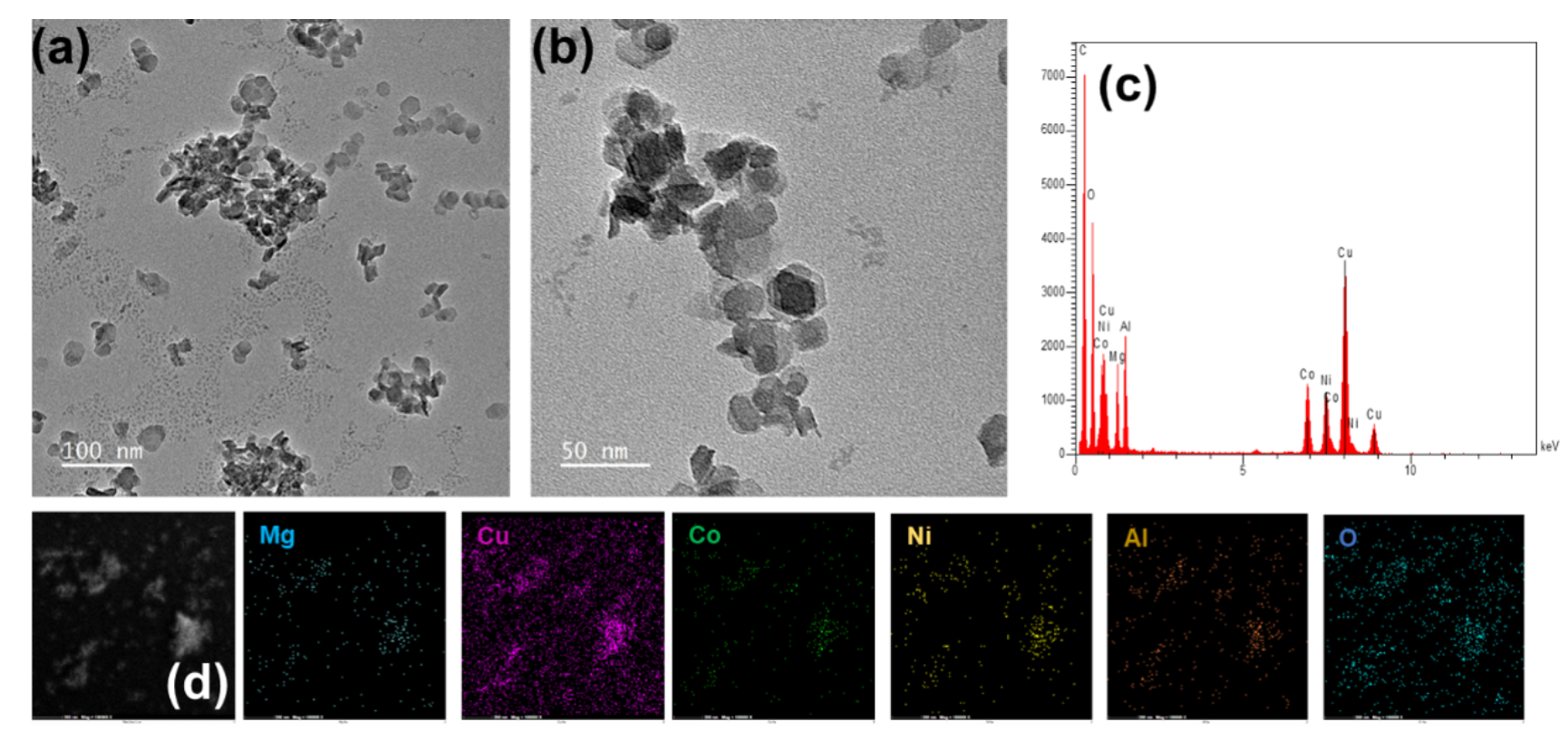


**Figure S3.** (a) TEM figure of (MgCuCoNi)_6_Al_2_-LDH (100 nm), (b) TEM figure of (MgCuCoNi)_6_Al_2_-LDH (50 nm), (c) and (d) The EDS mapping of (MgCuCoNi)_6_Al_2_-LDH


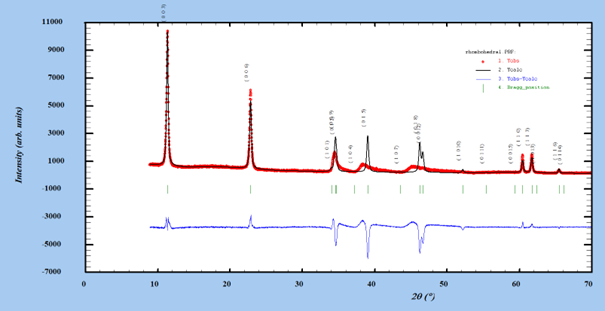


**Figure S4.** Refinement figure of Mg_6_Al_2_-LDH


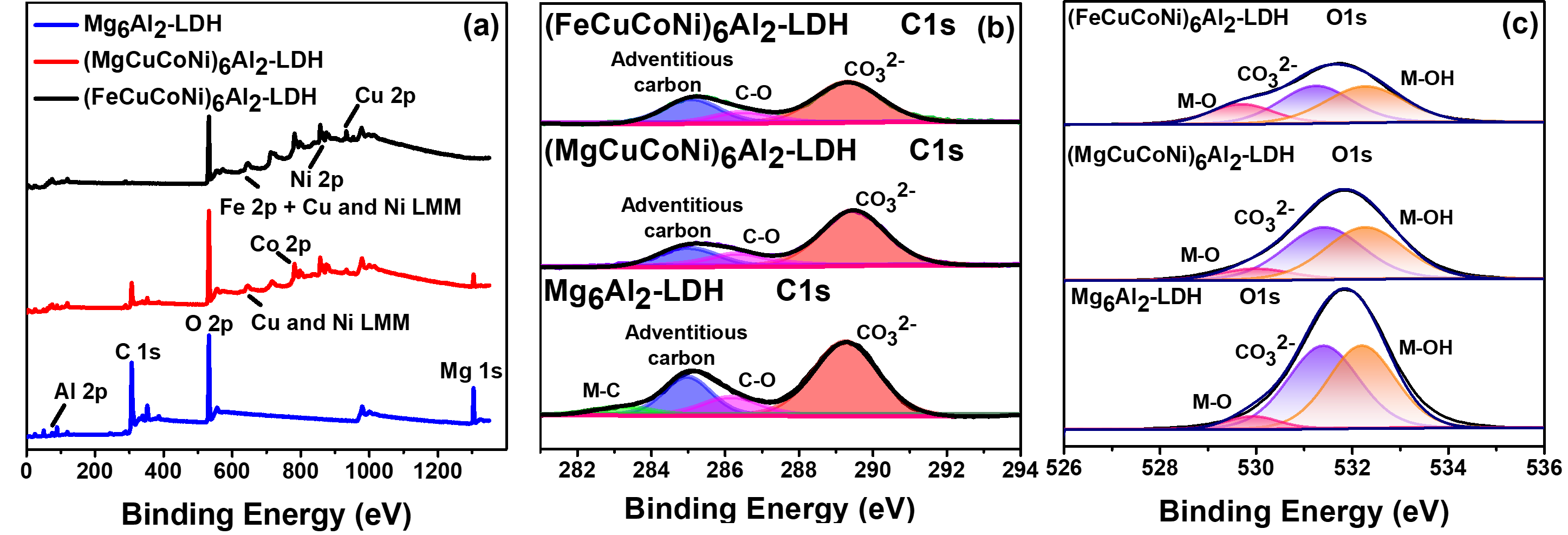


**Figure S5.** XPS spectra of (a) survey, (b) C1s peak spectra, (c) O 1s peak spectra of Mg_6_Al_2_-LDH, (MgCuCoNi)_6_Al_2_-LDH and (FeCuCoNi)_6_Al_2_-LDH


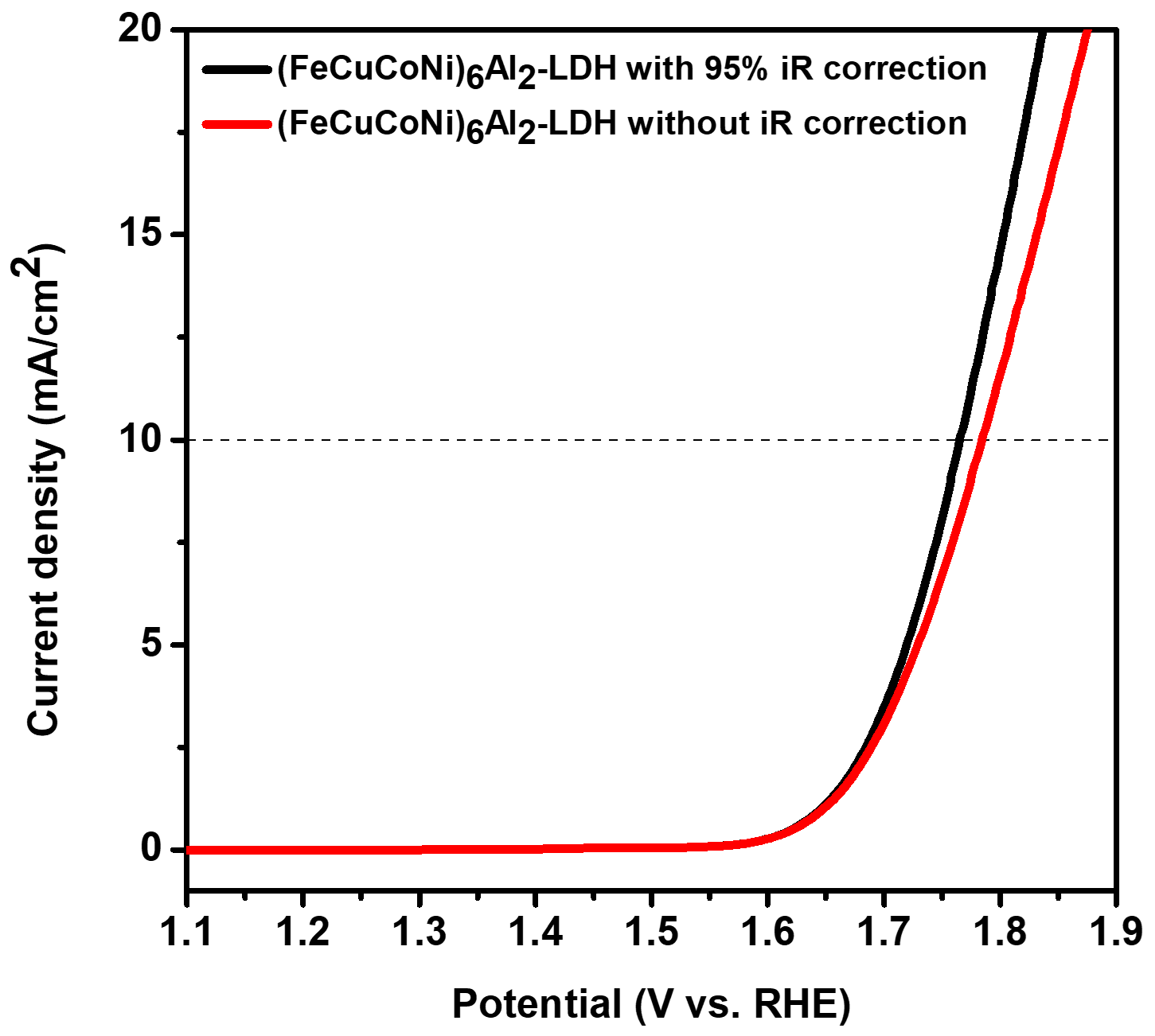


**Figure S6.** LSV curves (with 95% iR correction and without iR correction)


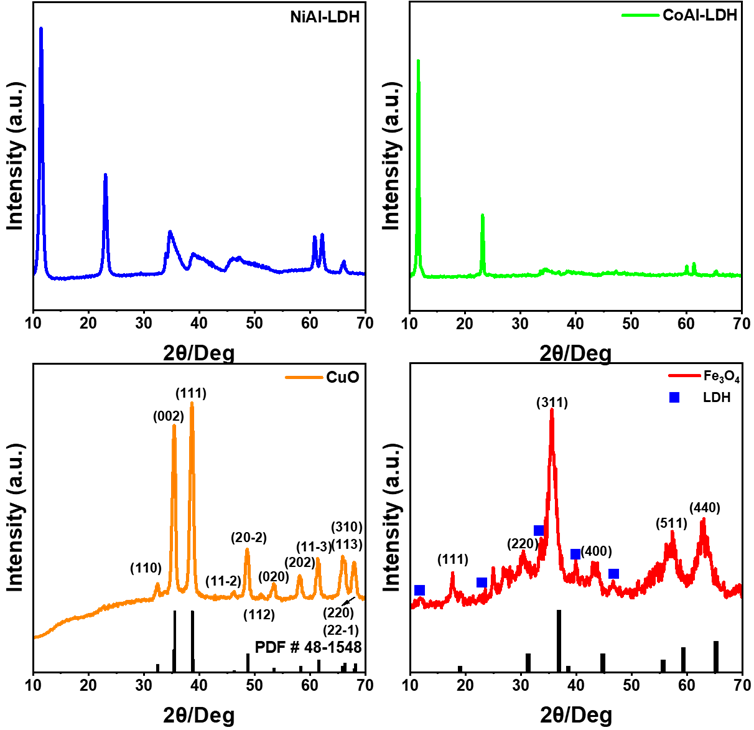


**Figure S7.** XRD patterns for samples with Mg substituted in the LDH structure by Ni, Co, Cu, and Fe.


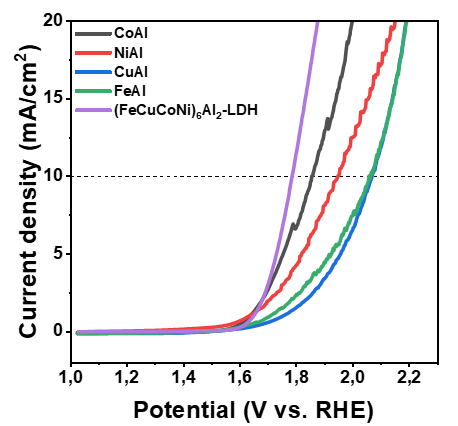


**Figure S8.** Linear sweep voltammetry for samples with Mg substituted in the LDH structure by Ni, Co, Cu, and Fe, and compared with (FeCuCoNi)_6_Al_2_-LDH.

**Table S2.** The R_ct_ value

| Sample | R_ct_ (kΩ) |
| --- | --- |
| Mg_6_Al_2_-LDH | 22.70 |
| (MgCuCoNi)_6_Al_2_-LDH | 26.57 |
| (Mg_2/3_Fe_1/3_CuCoNi)_6_Al_2_-LDH | 29.24 |
| (Mg_1/3_Fe_2/3_CuCoNi)_6_Al_2_-LDH | 6.82 |
| (FeCuCoNi)_6_Al_2_-LDH  IrO_2_ | 3.16  2.00 |


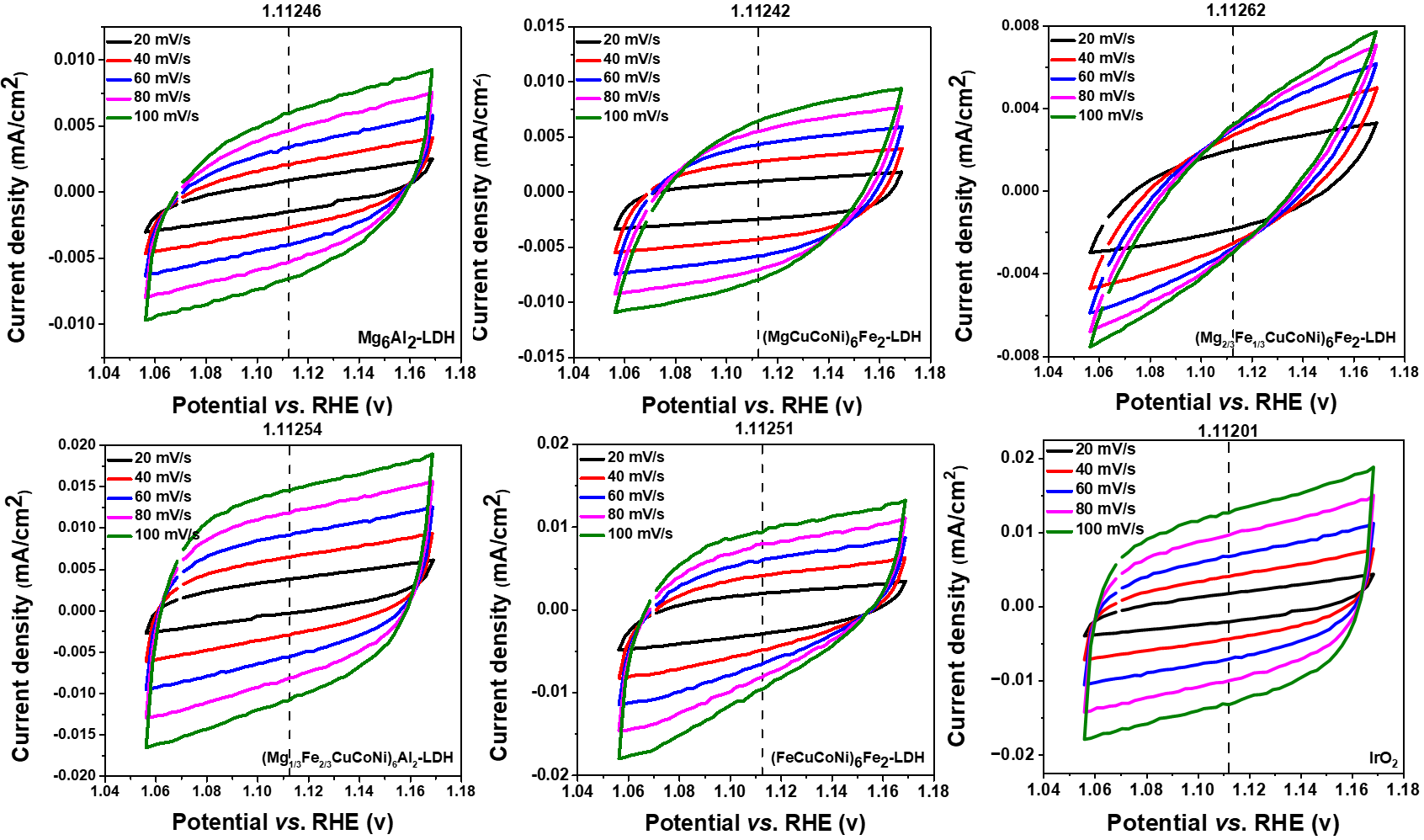


**Figure S9.** CV curves for ECSA calculation
